# Beyond the Very Important Pharmacogenes (VIPs): Uncovering Shadow Pharmacogenes in the Human Drug Response Network

**DOI:** 10.1021/acsomega.5c12757

**Published:** 2026-03-19

**Authors:** Nicolly Clemente de Melo, Guilherme Silva Accioli, Karen Sánchez-Luquez, Mateus Freitas de Farias Gomes, Aline Cristina Felicio, Lucas Miguel de Carvalho

**Affiliations:** 154623 São Francisco University, Bragança Paulista, São Paulo 12916-900, Brazil

## Abstract

Understanding the molecular determinants of interindividual drug response variability remains a major challenge in pharmacogenomics. Very Important Pharmacogenes (VIPs), as defined by PharmGKB, represent genes with well-established roles in drug metabolism and efficacy. However, their activity occurs within complex molecular networks that extend beyond direct pharmacogenetic associations. We constructed a VIP-centered subnetwork and applied network topology analyses, including shortest path, signal propagation, and degree centrality, to identify key nodes mediating VIP interactions. Functional enrichment, transcription factor (TF) association, and drug–gene interaction analyses were subsequently performed to characterize the biological and pharmacological context of these networks. Our results revealed a dense VIP interactome enriched in metabolic, endocrine, and signaling pathways. Notably, we identified a subset of highly connected non-VIP genes that frequently bridge canonical VIPs, termed shadow VIPs. These genes, often encoding transcriptional regulators, such as *NR1I2*, *NR1H4*, and *ESR2*, and more frequent in the shortest paths connecting VIPs, such as POR, APP, and GIPC1, exhibited strong associations with approved drugs, particularly hormone-related and antineoplastic agents. This suggests that shadow VIPs may act as indirect regulators of pharmacogenomic phenotypes by influencing the expression or activity of canonical VIPs. Additionally, the analysis revealed that shadow VIPs, on average, exhibit lower RVIS values than VIPs, indicating a higher intolerance to functional mutations. This suggests that shadow VIPs are under stronger selective selection, underscoring their essential biological roles. Together, these findings expand the current pharmacogenomic framework, demonstrating that drug response mechanisms emerge from a wider network of regulatory and functional interactions. Introducing the concept of shadow VIPs highlights new molecular candidates for pharmacogenetic exploration and emphasizes the value of network-based approaches in advancing precision medicine.

## Graphical abstract

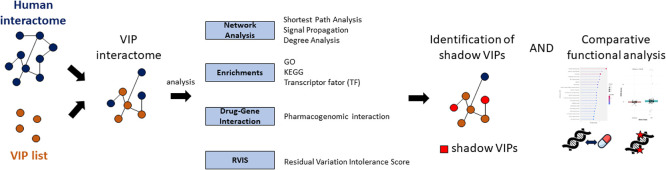

## 1 Introduction

Precision medicine aims to tailor therapeutic strategies according to individual biological variability, and pharmacogenomics has become a cornerstone of this paradigm.^1^ By customizing drug selection and dosage to align with a patient’s genomic characteristics, it strives to improve therapeutic outcomes and minimize adverse drug reactions (ADR). Genetic variation is estimated to account for up to 80% of interindividual differences in drug response in Sardinians and Zhuang populations.^2,3^ A recent research reveals more than 8 million new variants in Brazil’s population, which is significant due to genetic contribution of underrepresented populations to the formation of Brazilian genetic identity.^4^ Pharmacogenomics seeks to explain how inherited genetic variation contributes to heterogeneity in drug efficacy and ADRs, a leading cause of preventable morbidity and mortality worldwide.^2^ Variants such as single nucleotide polymorphisms (SNPs) contribute to phenotypic heterogeneity in drug response, emphasizing the clinical relevance of profiling gene-to-protein effects.^5^

By analyzing how drugs influence proteins and their interactions, pharmacoproteomics provides a deeper understanding of drug mechanisms and offers insights that can further optimize personalized medicine strategies.^6^ Protein–protein Interaction (PPI) networks are crucial for unraveling the complexity and dynamics of biological systems.^7^ These networks illustrate protein interactions that underpin diverse processes, including cellular communication and metabolic activities.^8^ Exploring these networks allows to reveal concealed protein relationships, offering valuable insights into intricate pathways and potential drug targets.

Within this field, substantial effort has focused on defining Very Important Pharmacogenes (VIPs) that encode proteins that play a role in the metabolism of many drugs (e.g., *CYP2D6*), or contain variants which potentially contribute to a severe drug response (e.g., *HLA-B*) (https://www.pharmgkb.org/vips). VIPs has strongest clinical and experimental evidence linking genetic variation to pharmacological outcomes.^9^ Currently, 34 VIPs have been listed including genes related to drug metabolism (Phase I and II) (*CYP* family, *COMT, DPYD, G6PD, GSTP1, MTHFR, NAT2, NUDT15, TPMT, TYMS, UGT1A1*), drug transport genes (*ABCB1, ABCG2, SLC19A1, SLCO1B1*), drug target genes (*ACE, ADRB1, ADRB2, DRD2, VKORC1*), immune pharmacogenomic genes (*HLA-B, IFNL3*), genes involved in cellular stress and mitochondrial function (*G6PD, MT-RNR1*) and ion channel, membrane physiology genes (*CFTR*, *CACNA1*S, *RYR1*). Over time, new genes are added to the VIP list as they are incorporated into clinical guideline recommendations by Clinical Pharmacogenetics Implementation Consortium, and existing entries are periodically reevaluated to reflect the evolving evidence base.^10^ This dynamic process highlights a critical window in which bioinformatics tools can play a major role, by identifying emerging pharmacogenes, reassessing variant-gene-drug interactions, and integrating network-based analyses to support guideline updates and clinical implementation.

While network medicine approaches have been applied to disease gene prioritization, their systematic application to pharmacogenomic VIP-centered networks remains limited. Most network-based frameworks have focused primarily on disease modules, drug repurposing, or global drug–target interactions, often emphasizing disease-associated genes rather than pharmacogenes with established clinical relevance. Recent advances in pharmacogenomics highlight the growing importance of integrating multiomics data, network-based analyses, and systems biology approaches to better capture the complex molecular interactions underlying interindividual variability in drug response.^11^ In this context, network analysis has been recognized as a critical tool for deciphering drug–gene interactions, regulatory mechanisms, and functional dependencies that extend beyond canonical pharmacogenes, enabling the identification of indirect regulators and modulators of pharmacokinetic and pharmacodynamic processes.^11,12^ However, despite the rapid expansion of network medicine in drug discovery and disease modeling, few studies have systematically anchored these approaches to curated pharmacogenomic resources such as PharmGKB VIPs, or explored how noncanonical genes may mediate drug response through their topological and regulatory proximity to established pharmacogenes.^12^ This gap underscores the need for pharmacogenomics-oriented network frameworks that integrate interactome topology, regulatory mechanisms, and pharmacological evidence to expand the current understanding of drug response variability.

Although the human interactome remains inherently incomplete, advances in network science have shown that robust biological inference can still be achieved through formal reconstruction methods capable of handling missing and uncertain interactions. These approaches support the use of curated but partial interaction networks to uncover meaningful biological patterns relevant to pharmacogenomics.^13,14^ Mapping VIPs within the interactome and characterizing their closest functional partners allows the identification of secondary or “shadow” pharmacogenes that may influence drug response through shared pathways, regulatory mechanisms, or signaling networks. Beyond the core pharmacogenes, this research focuses on the human interactome to uncover “Shadow” VIPs, secondary genes that interact directly with primary VIPs. We seek to define the functional landscape of these networks by integrating functional enrichment and drug–gene interaction data with analyses of gene intolerance to functional genetic variation. This approach allows us to characterize the biological/clinical relevance of these partners, providing insights into the molecular mechanisms underlying drug efficacy and toxicity.

## 2 Materials and Methods

Before diving into the identification of VIPs and subsequent analyses, we first established a clear workflow for the entire process, which is summarized in Figure 1. This pipeline outlines the construction of the human PPI network to identify “Shadow VIPs”. These secondary genes are subsequently characterized through functional enrichment, drug–gene interactions, and Residual Variation Intolerance Score (RVIS) assessments to elucidate their biological relevance within the VIP-centered interactome.

**Figure 1:**
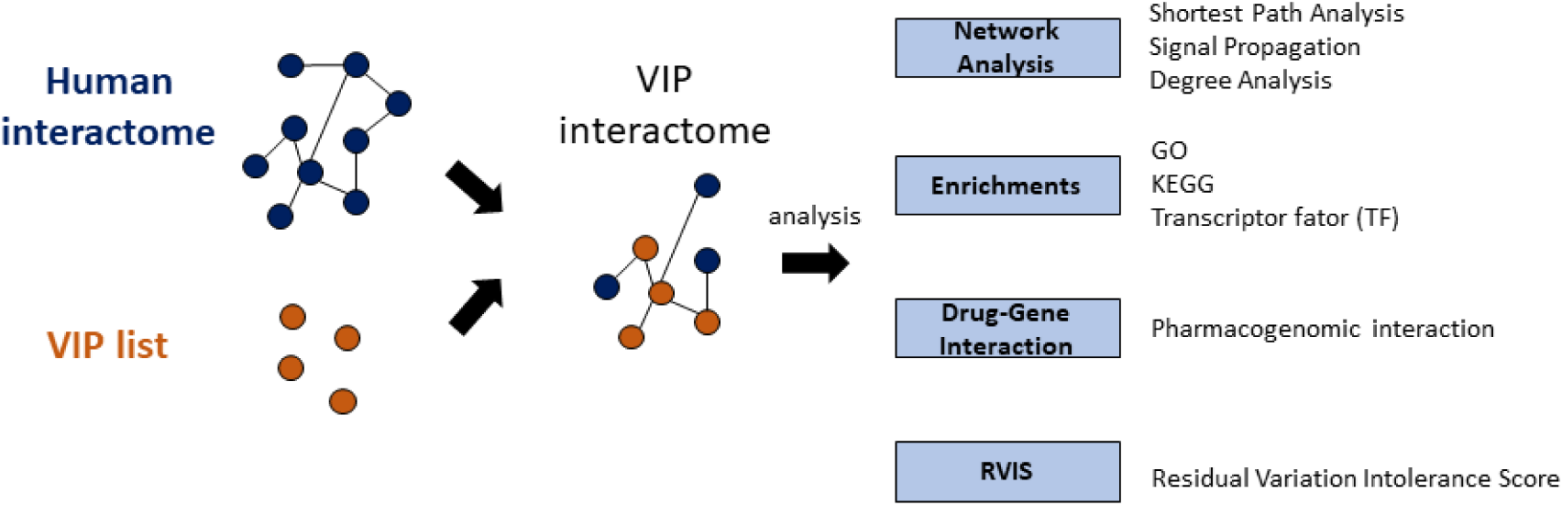
Schematic representation of the analytical workflow used to describe and explore the VIP-centered interactome. The figure illustrates the steps involved in constructing the human PPI network and performing subsequent descriptive analyses, including functional enrichment, drug–gene interaction, and RVIS analyses.

### 2.1 Identification of Very Important Pharmacogenes (VIPs)

To identify the Very Important Pharmacogenes (VIPs), we consulted the PharmGKB database, a comprehensive resource that catalogs genetic variations influencing drug responses. The database provides valuable information regarding pharmacogenetic associations, including the relationships between genes and their influence on drug efficacy and adverse drug reactions. The VIPs were obtained (Accessed on February 4, 2026) from the PharmGKB VIPs section (https://www.pharmgkb.org/vips) (Supplementary File 1).

### 2.2 Network Analysis Process

The network analysis was conducted in several steps to explore the interactions of the VIPs within the human interactome and to gain insights into their role in drug response (Figure 1). The following steps were taken to generate the PPI network and perform subsequent analyses:

#### 2.2.1 VIP Interactome Construction

We began by obtaining the human interactome from the BIOGRID database v4.4.240,^15^ which provides a comprehensive map of protein–protein interactions, derived from experimental data reported in peer-reviewed publications, which are manually curated by experts. After, using the list of VIPs, we performed a query on the BIOGRID interactome to retrieve all the first-degree neighbors of the VIPs. This approach allowed us to construct a subnetwork centered around the VIPs, focusing on the direct and indirect protein interactions that are potentially relevant for drug response mechanisms.

#### 2.2.2 Network Analysis and Visualization

The network analysis was performed using the networkX package^16^ in Python. The script used to perform the analysis is in the Supplementary File 1. Several analyses were conducted on the constructed network, including:

(1) Shortest Path Analysis, where we analyzed the shortest paths between VIPs and other nodes in the network to uncover key pathways and connections. Furthermore, in the analysis of shortest paths, we analyze the main hub genes that appear most in the paths;
(2) Signal Propagation, where signal propagation analysis was performed to model how signaling events might propagate through the network from the VIPs;
(3) Degree Analysis, where the degree centrality of nodes in the network was analyzed to identify the most influential proteins (i.e., the hubs) in the network. These hubs play critical roles in biological processes and may serve as important therapeutic targets. Genes with higher degree performed a new PPI network that was visualized using Cytoscape.^17^ STRING^18^ was used to analyze the combined score related to an evidence suggesting a functional link and specifically the value that suggests a functional link between proteins by experimental/biochemical data with “high”, “medium”, and “exploratory” levels of evidence. Likewise, Functional Enrichment was filtered by “Reference Publications (PubMed)” with selected metrics: (i) Group terms by: “similarity = > 0.8”; (ii) Sort terms by: “-log­(FDR) and (iii) Number of terms shown: “15”.

### 2.3 Functional Enrichment Analysis

In addition to the network analysis, we performed functional enrichment (KEGG pathways and GO Biological Process) analysis using DAVID.^19^ The p-value adjustment was applied using the FDR method (<0.05) to control for false discovery rates.

### 2.4 Transcription Factor Enrichment Analysis

Transcription factor (TF) enrichment analysis was performed using the ChEA3 tool.^20^ The input gene list was compared against multiple TF-target gene libraries, including ChIP-seq and coexpression data sets. Significance of overlap was calculated using Fisher’s Exact Test with a background of 20,000 genes. P-values were adjusted by the Benjamini-Hochberg method. TF rankings from different libraries were integrated using the MeanRank method, where lower scores indicate higher relevance.

### 2.5 Drug-Gene Interaction Analysis

Drug-gene interaction data were obtained from the Drug-Gene Interaction Database (DGIdb),^21^ a comprehensive resource that integrates curated drug and gene interaction information from multiple publications and expert sources. As input we consider a list of genes. For each interaction, regulatory approval status, indication, and an interaction score were retrieved. The interaction score was used to rank interactions and assess the confidence in the reported drug-gene relationships. For each gene, we only consider drugs that have been approved and have a score greater than zero. Data were filtered and analyzed to explore both approved and investigational drugs targeting genes.

### 2.6 Residual Variation Intolerance Score

The Residual Variation Intolerance Score (RVIS) is a gene-level assessment system designed to rank protein-coding human genes based on their intolerance to functional genetic variation. This system quantifies whether a gene contains more or less common functional genetic variation than statistically expected, considering the amount of presumably neutral variation present in that gene.

1 Negative RVIS values: Indicate that the gene has less common functional variation than predicted. These scores usually reflect the action of strong selective selection against functional variation, suggesting that the gene is highly intolerant to mutations. Genes with the lowest (most negative) RVIS values are considered the most intolerant.
2 Positive RVIS values: Indicate that the gene contains more common functional variation than predicted. These scores likely reflect the absence of selective selection, the presence of balanced selection, or positive selection, suggesting that the gene is highly tolerant to functional variation.

The RVIS values were retrieved from the Genic Intolerance Database.^22^ In the RVIS analysis, we observed the ancestral group contributions by gene from AFR (African), AMR (Admixed American), SAS (South Asian), EAS (East Asian), ASJ (Ashkenazi Jewish), and NFE (Non-Finnish European).

### 2.7 Identification of Shadow VIPs

In addition to the established set of Very Important Pharmacogenes (VIPs), our network-based approach revealed a subset of non-VIP genes that appear to play central roles in mediating interactions among VIPs and their functional modules. We propose to refer to these nodes as “shadow VIPs”, genes that, while not formally recognized as pharmacogenes, may exert significant influence on drug response mechanisms through their topological and regulatory connections.

By analyzing the interactome topology, we observed that several of these shadow VIPs occupy hub-like positions or serve as recurrent intermediates in the shortest paths between canonical VIPs. Importantly, such genes could modulate pharmacogenetic phenotypes indirectly, for instance by regulating the activity, expression, or stability of VIPs within shared signaling or metabolic contexts. Moreover, transcription factor enrichment analysis supports the existence of potential regulatory mechanisms involving both VIPs and other genes, highlighting the joint modulation and amplifying their impact on drug metabolism and response. We consider as shadow VIPs the top 10 most frequent genes among all the shortest paths connecting all VIP genes, together with the top 10 transcription factors that most actively regulate the VIPs.

## 3 Results and Discussion

### 3.1 VIPs’ Interactome

From the PharmGKB database, we identified 34 Very Important Pharmacogenes (VIPs) (Supplementary File 1). After constructing the subnetwork of the human interactome using the BIOGRID database, we generated a network consisting of 2,919 nodes and 157,842 edges. The global network metrics indicated a dense and highly connected structure, with a diameter of 5, a clustering coefficient of 0.256, network heterogeneity of 1.272, and an average number of neighbors of 107.503, highlighting the strong interconnection among the VIPs themselves.

The analysis of the network hubs based on degree revealed the main VIP genes, with the following degree values: *CFTR* (1558), *ADRB2* (327), *HLA-B* (246), *G6PD* (208), and *GSTP1* (208). Interestingly, when we examined the top 10 genes with the highest degree in the network (Supplementary File 1), only CFTR appeared among the most connected hub genes. The top 10 genes with the highest degree in the network were: *CFTR* (1558), *TRIM67* (1524), *ZRANB1* (1271), *PARK2* (1162), *RPA1* (1122), *RPA3* (1088), *RPA2* (1073), *MYC* (1067), *PLEKHA4* (1052), and *EGFR* (1004).

Additionally, the analysis of the shortest paths (SP) between all VIPs revealed the key genes involved in communication cascades between them (Table 1). We identified the main genes that appear most frequently in these pathways, highlighting their molecular functions, mechanisms related to drug resistance, and the affected drug classes. These genes play diverse roles, ranging from enzyme modulation and receptor signaling to epigenetic regulation and ion transport, ultimately impacting the pharmacokinetics and pharmacodynamics of various drugs.

**Table 1:** Most Frequently Genes Identified in VIPs Genes Shortest Path Analysis

Gene	Frequency	Molecular Function	Mechanism Related to Drug Resistance	Affected Drug Classes	Reference
*POR*	86	Transfers electrons from NADPH to cytochrome P450 microsomal enzymes, playing a role in the biotransformation of hormones, drugs, and xenobiotics.	Modulation of CYP enzyme activity, affecting drug metabolism.	Chemotherapeutics, anti-inflammatory drugs, hormone therapies.	Zhao et al. (2021)^23^
*APP*	35	Transmembrane glycoprotein is involved in the central nervous system, contributing to neuronal growth, cell adhesion, and copper homeostasis.	Interferes with the response to neuroprotective and antioxidant drugs.	Acetylcholinesterase inhibitors.	dos Santos et al. (2024)^24^
*GIPC1*	21	Involved in signaling mechanisms associated with G-protein, playing a role in regulating receptor trafficking and expression on the cell surface. Negative regulation of FGFR3 signaling and TGFBR3-mediated signaling are other relevant biological pathways.	Activation of cell survival pathways (PI3K/AKT and MAPK/ERK), conferring resistance to targeted therapies.	FGFR inhibitors.	Ahmed, Mythreye, and Lee (2021)^25^
*ISG15*	21	Plays a role in innate immune response against viral infections, acting either in a conjugated form (ISGylation) or as a free/unconjugated protein.	Modulation of inflammatory response and apoptosis, reducing the efficacy of antivirals and chemotherapeutics.	Interferons, cisplatin, protease inhibitors.	Sandy, Da Costa, and Schmidt (2020)^26^
*SEC23B*	21	Participates in vesicle formation in the ER, aiding in membrane deformation and cargo selection for transport to the Golgi complex.	Alteration in endocytosis and protein secretion, impacting drug absorption.	Monoclonal antibodies.	Rosato et al. (2022)^27^
*KIAA1429*	21	Regulates RNA methylation (m6A), influencing mRNA processing and splicing.	Epigenetic regulation affecting the expression of genes involved in apoptosis.	Methylation inhibitors.	Huang, Guo, and Jia (2024)^28^
*TRIM67*	21	Regulates protein localization and degradation, inhibits Ras protein signaling, and promotes neuronal development.	Regulation of protein stability involved in signal transduction.	Ras pathway inhibitors and neuroprotective agents.	Huang et al. (2022)^29^
*ATP2B3*	17	Transports calcium ions out of cells, maintaining intracellular calcium homeostasis.	Alteration in calcium signaling, reducing the efficacy of calcium-modulated drugs.	Calcium channel blockers.	Vallese et al. (2022)^30^
*ESR2*	16	Estrogen receptor involved in gene expression regulation, affecting cellular growth and reproductive, skeletal, cardiovascular, and nervous system functions.	Dysregulation of hormone response, leading to endocrine resistance.	Tamoxifen, aromatase inhibitors.	Gregorio, Laurindo, and Machado, (2021)^31^
*NTRK1*	15	Tyrosine kinase receptor activated by NGF, regulating survival, differentiation, and cell proliferation in the nervous system.	Activation of cell survival pathways, making cancer cells resistant.	Tyrosine kinase inhibitors.	Manea et al. (2022)^32^

#### 3.1.1 Degree Analysis

The proteins encoded by our genes of interest were categorized into two groups—“degree-all-genes”, including VIPs, and “degree-VIPs”. We identified the top 10 most connected proteins within each category based on the degree metric, which highlights nodes with the highest number of connections in the network.^33^ We refer to the top 10 by degree within the VIPs as “T10-Vip” and the top 10 by degree among all genes as “T10-All”.

At the Degree Analysis, our objective was to compare these groups to determine whether a relationship exists between non-VIP proteins and VIP-proteins and, if so, to elucidate their interconnections.

After merging T10-Vip with T10-All, the network constructed revealed *Cystic fibrosis transmembrane conductance regulator* (CFTR), a VIP gene, with the highest degree value in both categories, suggesting its role as the most important node in the network (Figure 2). The non-VIP proteins *PLEKHA4*, *ZRANB1*, and *TRIM67* are not directly related to the others, while the VIPs *COMT*, *VKORC1*, and *CYP2C9* do not make connections with the normal proteins with a higher degree. It remains for our analysis to understand what the levels of correlation between VIP’s and All Genes exist, which are truly connected.

**Figure 2:**
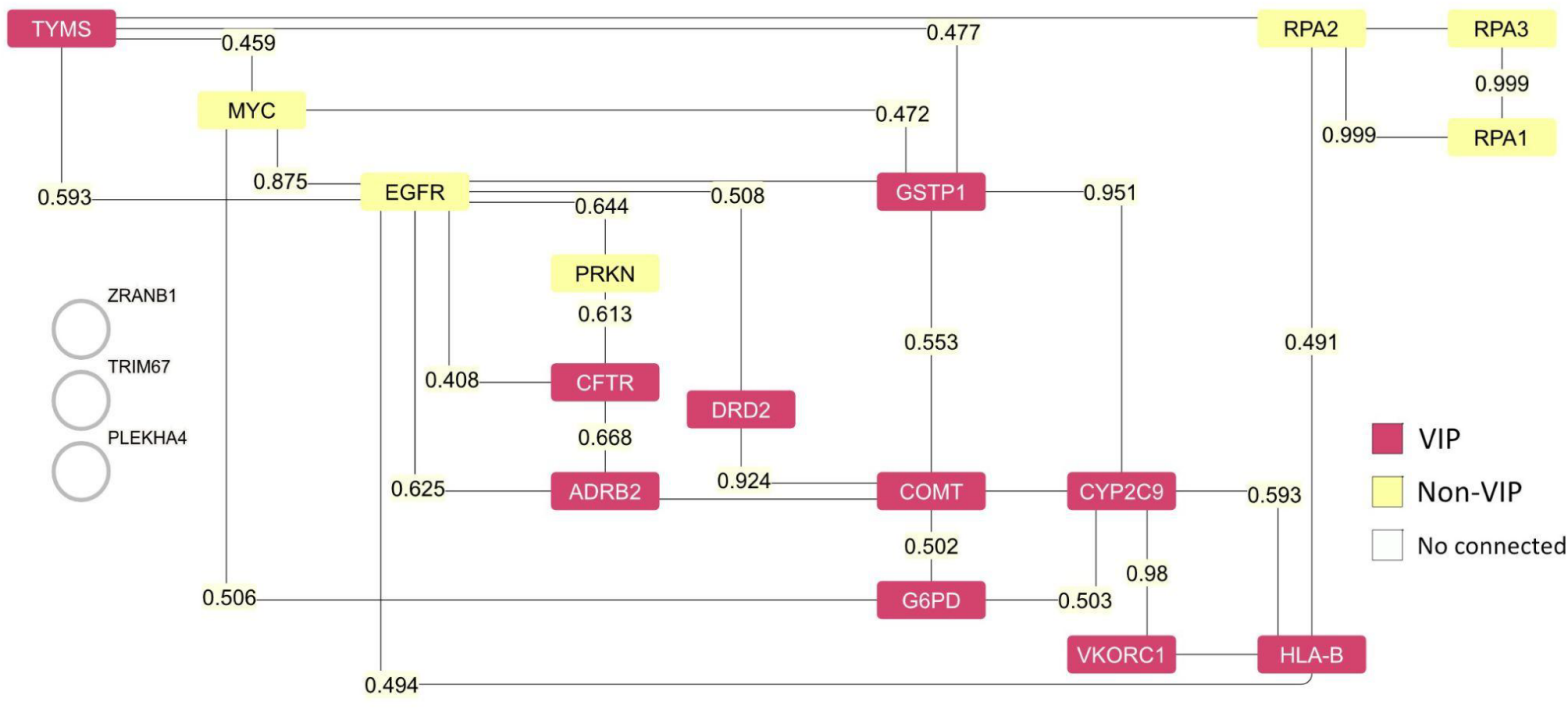
Protein–protein interaction network of T10-Vip and T10-All. The network illustrates interactions between proteins (nodes) derived from STRING database and visualized using Cytoscape. The network represents the union of the top 10 most connected proteins within T10-Vip and T10-All, based on their degree centrality. Pink nodes represent VIP proteins, while yellow nodes denote Non-VIP proteins. Edge labels indicate the combined score of the protein–protein interaction. Proteins shown as white circles (*ZBANB1*, *TRIM67*, *PLEKHA4*) are identified targets that did not show connections in this specific network.

From STRING, accessed on March 12, 2025, we considered only the high score of evidence suggesting a functional link and found that the *Epidermal Growth Factor Receptor* (*EGFR*) linked with *MYC Proto-Oncogen* (*MYC*) presents a high combined score of 0.875, but with an exploratory character of a laboratory test detecting protein–protein interaction by genetic interference assay.^34^ Looking further, we have found evidence that corroborates this connection: in oral carcinoma, *MYC* and *EGFR*, concomitant with other genes, present amplification in the number of copies.^35^

In the genomic evaluation of gallbladder adenocarcinoma, among the tumors that present *MYC* amplification, two tumor cell populations have the coexistence of amplified *MYC* and *ERBB2*, and *MYC* and *EGFR*, and it is concluded that genomic instability due to *MYC* amplification can cause specific *EGFR* and/or *ERBB2* amplification.^36^ The *EGFR*-*MYC*-*TXNIP* axis in Triple-negative breast cancer (TNBC) was modulated by silibinin, showing the importance of the *EGFR*-*MYC*-*TXNIP* axis in regulating TNBC metabolism.^37^

Another connection with a high combined score link was *EGFR*/*GSTP1*, with 0.713. In tumor cells, *GSTP1* is a downstream phosphorylation target of *EGFR*.^38^ Laboratory test presents three pieces of evidence of protein–protein interaction, two with medium confidence (by BIOGRID) detected by enzymatic study assay,^38^ and the other one detected by affinity chromatography technology assay.^39^ One with exploratory detection confidence (by IntAct) was detected by pull down assay.^40^

All these evidence show that attention has to be above the VIP proteins, but not exclusively. Non-VIP proteins play an important role in the development of disease and could represent additional therapeutic targets, as well as VIP. Functional enrichment based on literature, the reference publications sorted from PubMed relate the non-VIP *EGFR* present in 10 out of 15 articles (Supplementary File 2), reinforcing the importance of looking for the VIP-network interaction, measuring the impact on regulation by the presence of non-VIP protein.

### 3.2 KEGG Pathways Related to VIPs’ Interactome

We performed functional enrichment of the interactome generated by VIPs in intersection with the human interactome to better investigate the KEGG pathways and biological processes linked to it. Using the DAVID, we identified significant enrichment in pathways (Figure 3) associated with cell signaling, such as estrogen (hsa04915), HIF-1 (hsa04066), cAMP (hsa04024), mTOR (hsa04150), Ras (hsa04014), FoxO (hsa04068), and oxytocin signaling (hsa04921). Additional enrichment was observed in metabolic processes, including glycolysis/gluconeogenesis (hsa00010), amino acid biosynthesis (hsa01230), and cholesterol metabolism (hsa04979), as well as neurotransmission pathways, such as glutamatergic (hsa04724) and GABAergic synapses (hsa04727).

**Figure 3:**
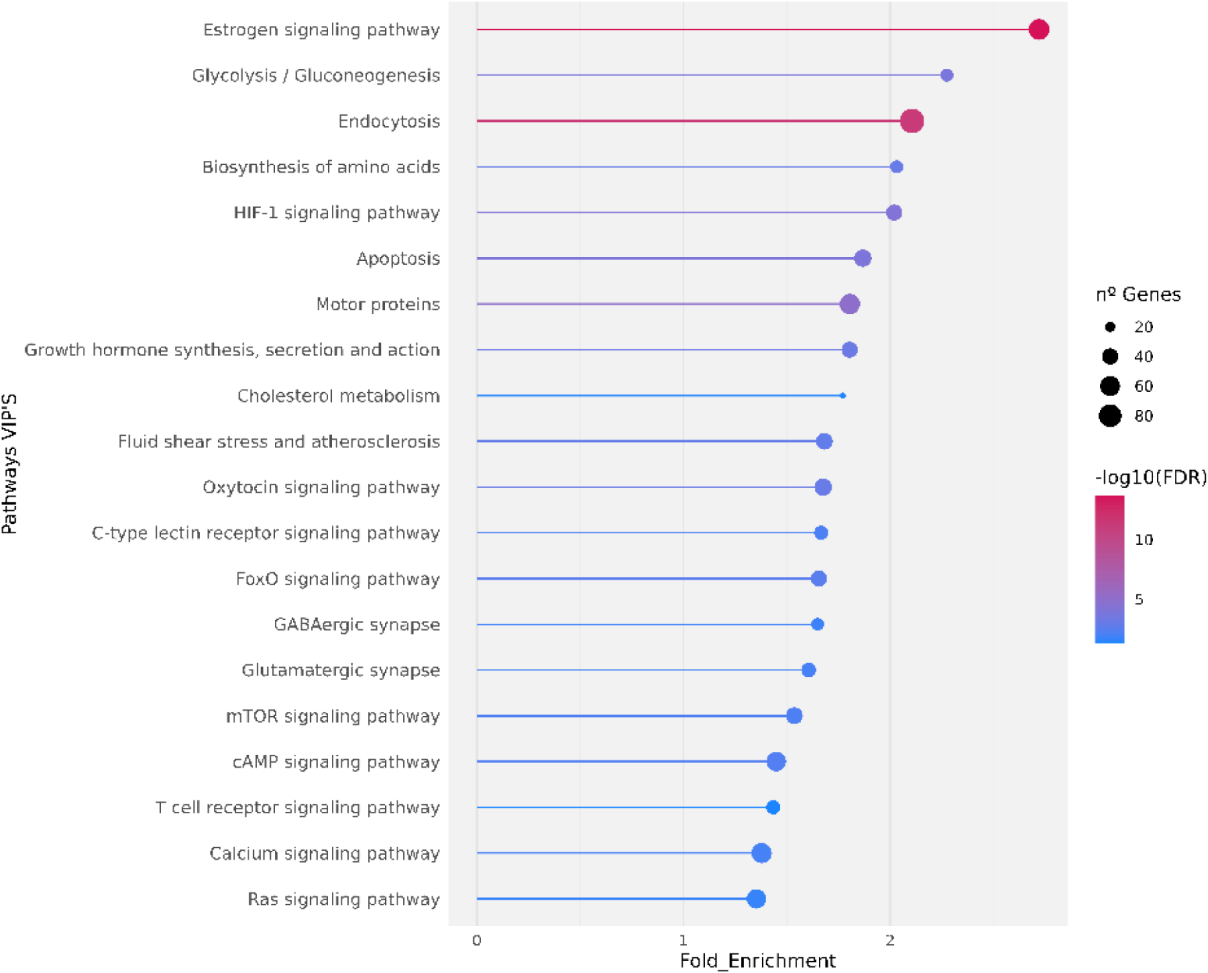
Functional enrichment pathways by KEGG for very important pharmacogenes (VIPs) interactome.

Several pathways identified play key roles in drug response and toxicity modulation. For instance, the apoptosis pathway (hsa04210) was enriched, reflecting the central role of programmed cell death in pharmacogenomic mechanisms and treatment sensitivity.^41,42^ Similarly, the FoxO signaling pathway participates in apoptosis, stress resistance, glucose metabolism, and drug resistance, supporting its importance in tumor suppression and therapeutic response.^43,44^ The HIF-1 pathway is another relevant finding, as it mediates cellular adaptation to hypoxia and contributes to drug resistance and cancer progression.^45,46^

In addition, pathways such as endocytosis (hsa04144) highlight mechanisms involved in drug internalization and delivery, essential for the development of targeted therapies.^47^ Motor proteins, which regulate intracellular transport along microtubules, also emerge as potential mediators of drug delivery efficiency.^48^ Altogether, these findings suggest that VIPs participate in a highly interconnected network of metabolic, signaling, and regulatory pathways that collectively shape interindividual variability in drug response.

### 3.3 Transcriptor Factor (TF) Enrichment Analysis

Transcription factor enrichment analysis using ChEA3 identified the top 10 candidate TFs potentially regulating the VIP gene set (Supplementary File 1). The highest-ranked TFs included *ARID3C*, *HNF1A*, and *NR1H4*, among others (Table 2). These TFs are known to play roles in the regulation of drug-metabolizing enzymes and transporters. For example, *HNF1A* regulates several cytochrome P450 enzymes implicated in drug metabolism.^49,50^ *NR1H4* is involved in bile acid signaling and gene modulation, which affect drug detoxification and transport.^51–53^ *NR1I2* and *PPARA* also appear as relevant TFs, both linked to the transcriptional regulation of numerous phase I and II drug-metabolizing enzymes.^54–58^ This enrichment supports a regulatory network involving these TFs in modulating pharmacokinetics-related genes.

**Table 2:** Top 10 TFs Enriched by ChEA3 in the Input VIPs’ Gene List

Rank	TF	Score	Libraries (Ranks)	Overlapping VIP genes
1	ARID3C	8.0	ARCHS4 Coexpression (7); GTEx Coexpression (9)	SLCO1B1, CYP2C9, CYP2A6, CYP2C8, CYP2B6, CYP2D6, NAT2, CYP4F2, CYP2C19
2	HNF1A	13.33		CYP2C9, CYP2A6, CYP2C8, ABCB1, ACE, CYP2D6, NAT2, CYP4F2, CYP2C19, CYP3A5, IFNL3, CFTR
3	NR1H4	14.33	ARCHS4 (10); Enrichr Queries (13); GTEx (20)	SLCO1B1, CYP2C9, CYP2C8, ABCB1, CYP2B6, CYP2D6, NAT2, CYP4F2, DPYD, CYP3A4, ABCG2
4	MYRFL	20.0	ARCHS4 (15); GTEx (25)	ACE, CYP4F2, CYP3A4, CYP3A5, ABCG2
5	NR1I2	21.0	Literature ChIP-seq (81); ARCHS4 (1); Enrichr Queries (1); GTEx (1)	ABCB1, ACE, UGT1A1, CYP4F2, ADRB2, CYP3A4, CYP2C19, COMT, CYP3A5, SLCO1B1, CYP2C9, CYP2A6, CYP2C8, CYP2B6, CYP2D6, NAT2, DPYD, CFTR, ABCG2
6	PPARA	26.67	ARCHS4 (43); Enrichr Queries (14); GTEx (23)	CYP2C9, ABCB1, CYP2D6, NAT2, CYP4F2, DPYD, ADRB1, ADRB2, CYP3A5
7	CREB3L3	37.0	ARCHS4 (4); Enrichr Queries (88); GTEx (19)	SLCO1B1, CYP2C9, CYP2C8, ABCB1, CYP2B6, ACE, UGT1A1, CYP2D6, NAT2, CYP4F2, CYP3A4, ABCG2
8	NR1I3	39.67	ARCHS4 (11); Enrichr Queries (98); GTEx (10)	SLCO1B1, CYP2C9, CYP2A6, CYP2C8, CYP2B6, UGT1A1, CYP2D6, NAT2, CYP4F2, CYP3A5, ABCG2
9	MYOD1	41.8	ARCHS4 (54); ENCODE ChIP-seq (8); Enrichr Queries (38); ReMap ChIP-seq (55); GTEx (54)	RYR1, VKORC1, TPMT, GSTP1, CACNA1S, ADRB2, COMT, NUDT15, ABCG2
10	YBX3	45.0	ARCHS4 (50); GTEx (40)	RYR1, CACNA1S

### 3.4 Drug-Gene Interaction Analysis of VIPs

The analysis of VIPs through the DGIdb platform revealed drug-gene interactions, highlighting both approved and nonapproved drugs targeting these genes. Table 3 summarizes the number of approved and nonapproved drugs for each VIP gene, along with the maximum and minimum interaction scores of approved drugs. These scores reflect the strength and evidence behind each drug-gene interaction, with higher scores indicating stronger support and more reliable interactions. All drugs and its indications related to each VIP is summarized in Supplementary File 1.

**Table 3:** Drug Interactions for VIP Genes^a^

gene	Approved	Not Approved	max_score	min_score
ABCB1	158	77	0.4442876473	0.002922945048
ABCG2	49	15	0.5437895683	0.01418581483
ACE	42	17	4.424050725	0.01988337405
ADRB1	69	19	1.779674951	0.01913628979
ADRB2	85	44	0.809361218	0.009411176953
CACNA1S	39	14	0.8954339375	0.01758888092
CFTR	16	29	10.44075971	0.0351540731
COMT	35	7	2.485895169	0.02180609798
CYP2A6	18	4	7.250527578	0.05717831167
CYP2B6	51	13	0.4078421762	0.007415312295
CYP2C19	224	260	0.1078590879	0.001365304911
CYP2C8	17	5	1.016957115	0.04091206784
CYP2C9	225	208	0.1808445678	0.001526114496
CYP2D6	328	266	0.2197129569	0.0009060328119
CYP3A4	397	497	0.1167870214	0.0007391583632
CYP3A5	88	2	0.5800422062	0.01054622193
CYP4F2	6	5	2.636555483	0.2433743523
DPYD	10	46	0.4661053443	0.01635457348
DRD2	131	123	0.6165802979	0,001787189269
G6PD	79	21	2.923412719	0.007054567373
GSTP1	40	20	0.8700633093	0.007909666448
HLA-B	32	6	2.497789405	0.03270914697
IFNL3	16	9	10.44075971	0.0652547482
MTHFR	35	2	2.821826949	0.01216304719
NAT2	28	20	0.7250527578	0.01121215605
NR4A2	0	4	NA	NA
NUDT15	4	0	4.350316547	0.5800422062
RYR1	12	2	51.52582715	0.0981274409
SLC19A1	16	2	1.657263446	0.02636555483
SLCO1B1	53	16	0.7565767907	0.006877970825
TPMT	14	0	7.457685508	0.05038976695
TYMS	33	16	1.065383644	0.009685305855
UGT1A1	36	4	0.8700633093	0.01763641843
VKORC1	8	2	6.960506475	0.360026197

a The table shows the number of approved and non-approved drugs for each VIP gene, Along with the max_score and min_score of approved drugs, representing the highest and lowest interaction strengths, reflecting the evidence supporting the drug-gene interaction.

Notably, *CYP3A4* displayed the highest number of drug interactions, with 397 approved and 497 nonapproved drugs, showcasing its central role in pharmacogenomics. It also had a wide range of interaction scores, with a maximum score of 0.1167 and a minimum of 0.0007, reflecting varying levels of evidence across interactions. In contrast, *RYR1* has an exceptional maximum interaction score of 51.53, attributed to its strong association with approved skeletal muscle relaxants and inhalation anesthetics. Also, *CYP4F2*, *NUDT15*, and *VKORC1* had a smaller pool of drugs, but their interactions provide potential targets for further exploration. This data underscores the varying levels of clinical evidence and potential therapeutic relevance of VIP genes.

We also observed the most frequent indications of each interaction per gene, considering only interactions with approved drugs and a score greater than zero, with the analysis of approved drugs interacting with VIP genes highlighting a wide range of therapeutic areas. Among the most frequently represented drug indications are antineoplastic agents, analgesics, antipsychotic, and antihypertensive agents, which appear prominently in the interaction profiles of several VIP genes (Supplementary File 1).

Antineoplastic agents, in particular, are heavily represented, appearing as the top indication across multiple VIP genes. The role of pharmacogenes in the metabolism of chemotherapeutic drugs shows the importance of personalized medicine in oncology, as individual genetic variations can significantly impact drug metabolism and therapeutic outcomes. Similarly, analgesics and nonsteroidal anti-inflammatory drugs are frequently associated with VIP genes involved in pain management and inflammation. Antihypertensive agents also emerge as a significant category, reflecting the role of VIPs in regulating vascular function and response to cardiovascular drugs. Interestingly, drugs for antipsychotic and antidepressant treatment are also prominently featured, highlighting the importance of pharmacogenomics in psychiatric medicine. As drug-gene interactions are better understood, precision medicine can better address the variability in drug responses, improving the safety and efficacy of treatments for conditions such as cancer, pain, hypertension, and mental health disorders.

### 3.5 Drugs Interaction with “Shadow VIPs”

We decided to investigate the drugs previously reported for the shadow VIPs, defined as the key genes identified in the shortest path analysis and the main transcription factors found to be associated with the VIPs (Tables 1 and 2). The exploration of drug associations for the 20 shadow VIPs using the DGIdb database revealed that 12 of these genes are already linked to at least one approved drug (Table 4). This finding underscores the potential pharmacological relevance of genes not traditionally classified as VIPs, yet occupying key positions in the VIP-centered network.

**Table 4:** Drug Interactions for Shadow VIP Genes^a^

gene	Approved	Not Approved	max_score	min_score
APP	15	51	3.163866579	0.0173838823
ATP2B3	1	7	0.38385146	0.38385146
ESR2	50	36	0.6070209135	0.007313504982
HNF1A	1	1	2.175158273	2.175158273
ISG15	1	0	0.8286317232	0.8286317232
MYOD1	9	7	1.087579137	0.04409104608
NR1H4	17	52	2.648018767	0.005112005343
NR1I2	94	19	2.309902591	0.003142724614
NR1I3	11	3	0.6779714099	0.1347774489
NTRK1	23	48	1.102897153	0.006393606682
POR	10	0	1.740126619	0.1898319948
PPARA	21	45	0.988708306	0.03295694353

a The table shows the number of approved and non-approved drugs for each VIP gene, Along with the max_score and min_score of approved drugs, representing the highest and lowest interaction strengths, reflecting the evidence supporting the drug-gene interaction.

Among these genes, *NR1I2* and *ESR2* exhibited the highest number of approved drug associations, with 94 and 50 drugs, respectively. Both encode nuclear receptors (*Pregnane X Receptor* (PXR) and *Estrogen Receptor Beta*) that function as major transcriptional regulators of drug-metabolizing enzymes and transporters.^59,60^ Similarly, *NR1H4* (FXR) and *PPARA* showed multiple approved drug associations, reinforcing their role in metabolic and xenobiotic regulation pathways. Other shadow VIPs, such as *NTRK1* and *APP*, presented a considerable number of drug interactions, many of which correspond to targeted therapies or neuroactive compounds. This suggests that network proximity to VIPs may reveal genes involved in drug response mechanisms within specific physiological or disease contexts. Conversely, genes like *HNF1A*, *ATP2B3*, and *ISG15* displayed only one or few approved drug associations, which may indicate either limited pharmacological exploration or context-dependent relevance. The diversity of maximum and minimum interaction scores (ranging from 0.003 to 3.16) reflects the variability in evidence supporting these associations. Importantly, the presence of high-confidence interactions for several shadow VIPs suggests that their influence on pharmacological processes is not merely theoretical but supported by experimental or clinical data.

To further characterize the pharmacological context of the drugs targeting shadow VIPs, we analyzed the frequency of therapeutic classes associated with these interactions (Supplementary File 1). The most prevalent categories were hormone replacement agents (n = 59) and hormonal antineoplastic agents (n = 58), followed by *antineoplastic agents* (n = 25). This predominance of hormone-related and anticancer drugs is consistent with the functional nature of several shadow VIPs, such as *ESR2*, *NR1I2*, *NR1H4*, and PPARA, which encode nuclear receptors or transcriptional regulators central to metabolic and endocrine signaling.

These findings suggest that shadow VIPs may participate in pathways frequently modulated during cancer therapy and endocrine regulation, potentially influencing individual variability in drug efficacy or toxicity. The enrichment of *antineoplastic* and *hormonal* compounds also supports the hypothesis that pharmacogenomic regulation extends beyond direct drug-metabolizing enzymes to include upstream regulatory elements that modulate cellular response to hormone- or receptor-driven treatments.

Other recurrent drug categories, including antidiabetic agents (n = 17), antihypertensive agents (n = 21), and antithrombotic agents (n = 30), indicate broader metabolic and cardiovascular relevance of these shadow VIPs. This observation aligns with the known roles of nuclear receptors and metabolic regulators in lipid and glucose homeostasis, as well as in vascular function.

### 3.6 Residual Variation Intolerance Score

Among all genes analyzed by ancestrality, the highest RVIS values (Supplementary File 1), indicating the greatest tolerance to functional variation, were consistently observed for *HLA-B* across all ancestral groups. Conversely, the lowest RVIS values, reflecting the strongest intolerance to functional variation, were found for *RYR1*, particularly in the South Asian (SAS) population. In AFR, *HLA-B* showed the highest RVIS (10.609), whereas *ATP2B3* exhibited the lowest (−2.124). In EAS, *HLA-B* had the highest RVIS (18.610), while *RYR1* showed the lowest (−4.910). In ASJ, the most tolerant gene was again *HLA-B* (18.610), and the most intolerant was *RYR1* (−5.655). In NFE, *HLA-B* reached the highest RVIS (18.363), whereas *RYR1* had the lowest (−5.787). In SAS, *HLA-B* displayed the highest RVIS (14.875), while *RYR1* presented the lowest (−7.317). This pattern is biologically expected, as HLA genes are highly polymorphic and subject to balancing selection due to their immune function, in contrast to evolutionarily constrained genes such as RYR1 that are involved in core cellular processes. These findings provide a population-level framework for interpreting gene relevance beyond pharmacogenomic annotation alone.

When the results were stratified by gene class, VIP genes and shadow VIPs exhibited distinct RVIS patterns. Across all populations, *HLA-B* displayed the highest RVIS among VIPs (range: 10.6–18.6), confirming its exceptional genetic tolerance. The most intolerant VIP gene varied slightly by ancestry but was consistently among *RYR1* (SAS = −7.317), *CYP2D6* (SAS = −1.122), and *UGT1A1* (SAS = −0.580). Within the shadow VIP genes, the most variation-tolerant gene was *NTRK1* (EAS = 0.941), whereas the most intolerant was *ATP2B3* (AFR = −2.124).

The analysis revealed that shadow VIPs have, on average, lower RVIS values compared to VIPs, indicating that they are less tolerant to functional mutations (Figure 4). This higher intolerance suggests that these genes are under stronger selective selection, reflecting their crucial biological roles. Shadow VIPs are involved both in the regulation of VIPs, since many act as transcription factors, and as key components within the shortest paths connecting VIPs in interaction networks. When considered together with the network topology, these findings can be interpreted as a possible functional interrelationship between Shadow VIPs and VIPs, which may reflect their involvement in key biological processes related to mechanisms associated with VIPs.

**Figure 4:**
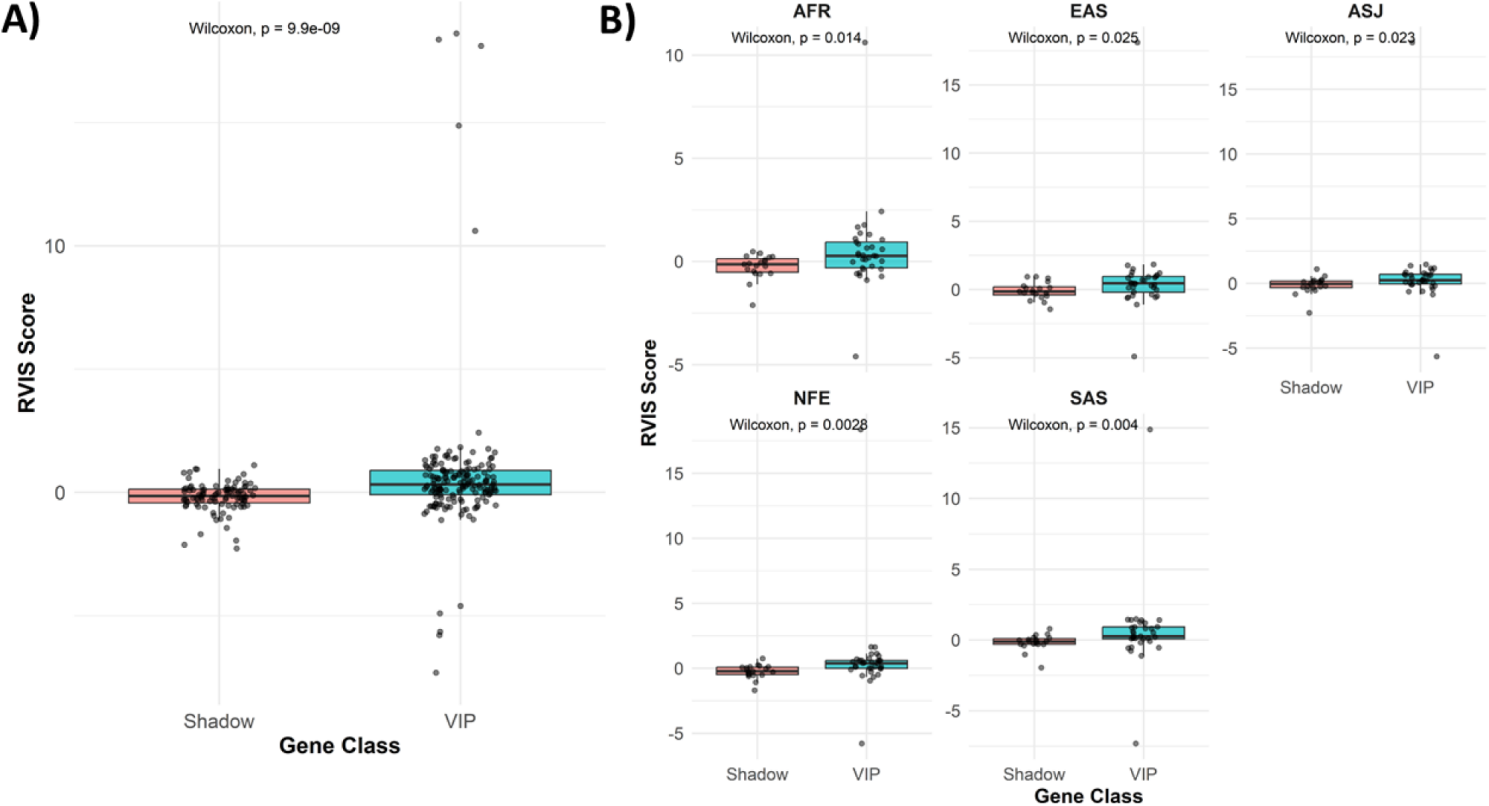
RVIS comparison by ancestry and gene class. (A) Global comparison of RVIS values between VIP and Shadow genes, regardless of ancestry. (B) Comparison of RVIS values between VIP and Shadow genes across different ancestral populations: AFR (African), EAS (East Asian), ASJ (Ashkenazi Jewish), NFE (Non-Finnish European), and SAS (South Asian). Statistical differences were assessed using the Wilcoxon test, and comparisons with *p* < 0.05 were considered statistically significant.

## 4 Conclusions

Our findings reveal a complex and interconnected pharmacogenomic landscape, where canonical Very Important Pharmacogenes (VIPs) interact with a broader network of regulatory and functional partners. By integrating network topology, transcription factor enrichment, and drug–gene interaction data, we uncovered a subset of highly connected non-VIP genes, designated as shadow VIPs, that appear to indirectly modulate pharmacogenomic pathways. These genes encompass diverse biological pathways relevant to pharmacogenomics, metabolic regulation, and cellular homeostasis. Genes such as *POR*, *NR1I2*, *NR1I3*, *PPARA*, and *CREB3L3* are key regulators of drug metabolism and lipid homeostasis, potentially modulating the expression and activity of cytochrome P450 enzymes and other xenobiotic-processing genes. Nuclear receptors including *ESR2*, *NR1H4*, and *HNF1A* participate in hormonal signaling, bile acid regulation, and glucose metabolism, linking endocrine control with pharmacokinetic variability. Several genes, including *ISG15*, *MYOD1*, *TRIM67*, and *YBX3*, contribute to immune responses, stress adaptation, and cellular differentiation. *ATP2B3* and *SEC23B* are involved in calcium transport and vesicular trafficking within the endoplasmic reticulum, supporting protein secretion and homeostasis. Additionally, *APP*, *NTRK1*, *ARID3C*, and *GIPC1* play roles in neuronal development and signaling pathways, while *KIAA1429* and *MYRFL* are implicated in RNA methylation and transcriptional regulation. Collectively, these genes illustrate the intricate interplay between metabolic, immune, and neuroendocrine networks that influence drug response and physiological adaptation.

The prevalence of hormone-related and antineoplastic compounds among shadow VIP–associated drugs underscores the importance of these regulatory nodes in mediating systemic drug responses. These results expand the conventional definition of pharmacogenes, highlighting that drug response variability may arise not only from direct drug-metabolizing enzymes but also from upstream regulators that influence their expression and activity.

The lower RVIS scores observed in shadow VIPs highlight their greater sensitivity to genetic variation and their potential importance in maintaining the stability of drug response networks. Given their central role in regulating VIP expression and mediating interactions between these genes, mutations in shadow VIPs could contribute to interindividual differences in drug efficacy or adverse reactions. These findings underscore the relevance of considering shadow VIPs in pharmacogenomic studies and in the identification of genetic factors influencing therapeutic outcomes.

Altogether, this study provides a systems-level framework for pharmacogenomic analysis, introducing the concept of shadow VIPs as integrative components of the drug response network. Recognizing these indirect regulators may improve our understanding of interindividual variability in therapy outcomes and guide the identification of novel biomarkers and targets for precision medicine.

It is important to discuss the recent advances in network science that have introduced mathematically grounded frameworks that extend beyond classical topological metrics to better capture the organization, robustness, and multiscale structure of biological networks. Several advanced network-based strategies have been proposed to address key challenges such as community detection in incomplete biological networks and the identification of critical nodes and multiscale structural properties, offering complementary perspectives beyond classical topological analyses (Artigo 1; Artigo 2). Although these advanced methodologies were beyond the scope of the present study, their future integration with pharmacogenomic and VIP-centered network analyses may provide complementary insights into network dynamics, resilience, and modular organization, further advancing systems-level interpretations of drug response variability.

We have some limitations in our study. First, our analysis was limited to a select group of 34 VIP genes. While these genes are well-curated, this small sample size may not fully capture the pharmacogenomic landscape. Second, topological parameters are essential but sensitive criteria in network methodology. Since there is no universally predefined set of metrics, their selection can significantly influence results. In this study, we chose our parameters based on clear biological rationale and literature evidence. However, we acknowledge that using a different set of metrics could provide different insights. Finally, as with most network-based approaches, our findings may be influenced by highly connected nodes that frequently appear in large interactome data sets. While these genes reflect central network properties, their specific pharmacogenomic relevance should be interpreted with caution as their prominence may stem from general systemic centrality rather than exclusive drug-related roles.

## Supplementary Material





## Notes

Supplementary File 1, complete data set generated in this study (XLSX)
Supplementary File 2, Figure S1 showing pharmacogenomic reference publications, PubMed enrichment, and VIP/non-VIP protein network mapping (PDF)
