# Supplementary material for: Beyond the Very Important Pharmacogenes (VIPs): Uncovering Shadow Pharmacogenes in the Human Drug Response Network
Source: ACS Omega. 2026 Mar 19;11(12):19333–44. doi: 10.1021/acsomega.5c12757 (PMC13044611; doi:10.1021/acsomega.5c12757)
Supplement: Supplementary file 2 [file ao5c12757_si_002.pdf]

Supplementary Material

Beyond the Very Important Pharmacogenes (VIPs): uncovering shadow pharmacogenes in the human drug response network

Nicolly Clemente de Melo[a, †], Guilherme Silva Accioli[a, †], Karen Sánchez-Luquez[a], Mateus Freitas de Farias Gomes[a], Aline Cristina Felicio[a], Lucas Miguel de Carvalho[a,*]

[a] São Francisco University, Bragança Paulista 12916-900, São Paulo, Brazil

*Email: lucas.miguel@usf.edu.br

† N.C.M and G.S.A contributed equally to this work

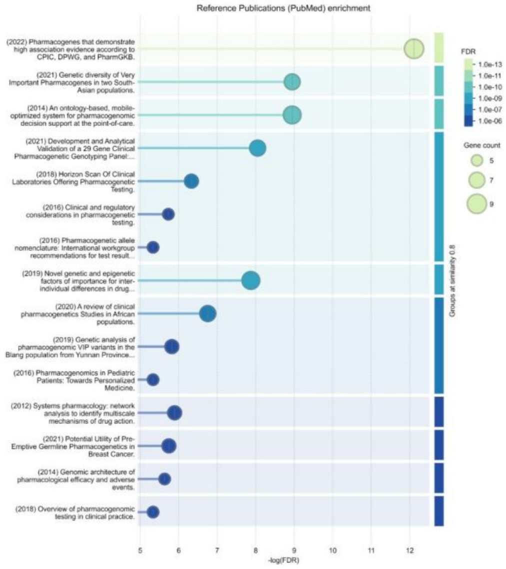

Term Description (Year ↑)	Matching Proteins From Network								
(2022) Pharmacogenes that demonstrate high association evidence according to CPIC, DPWG, and PharmGKB.	CFTR	CYP2C9	EGFR	TYMS	COMT	DRD2	VKORC1	GSTP1	HLA-B
(2021) Potential Utility of Pre-Emptive Germline Pharmacogenetics in Breast Cancer.	CFTR	CYP2C9	EGFR	COMT	DRD2	VKORC1	GSTP1		
(2021) Genetic diversity of Very Important Pharmacogenes in two South-Asian populations.	CYP2C9	ADRB2	TYMS	COMT	DRD2	VKORC1	GSTP1	HLA-B	
(2021) Development and Analytical Validation of a 29 Gene Clinical Pharmacogenetic Genotyping Panel: Multi-Ethnic Allele and Copy Number Variant Detection.	CFTR	CYP2C9	EGFR	COMT	DRD2	VKORC1	HLA-B		
(2020) A review of clinical pharmacogenetics Studies in African populations.	CFTR	CYP2C9	EGFR	ADRB2	TYMS	VKORC1	GSTP1	HLA-B	
(2019) Novel genetic and epigenetic factors of importance for inter-individual differences in drug disposition, response and toxicity.	CFTR	CYP2C9	ADRB2	COMT	DRD2	VKORC1	GSTP1		
(2019) Genetic analysis of pharmacogenomic VIP variants in the Blang population from Yunnan Province of China.	CFTR	CYP2C9	EGFR	COMT	VKORC1	HLA-B			
(2018) Overview of pharmacogenomic testing in clinical practice.	CYP2C9	EGFR	G6PD	VKORC1	HLA-B	MYC			
(2018) Horizon Scan Of Clinical Laboratories Offering Pharmacogenetic Testing.	CYP2C9	ADRB2	COMT	DRD2	VKORC1	GSTP1			
(2016) Pharmacogenomics in Pediatric Patients: Towards Personalized Medicine.	CYP2C9	COMT	G6PD	VKORC1	GSTP1	HLA-B			
(2016) Pharmacogenetic allele nomenclature: International workgroup recommendations for test result reporting.	CFTR	CYP2C9	EGFR	VKORC1	HLA-B				
(2016) Clinical and regulatory considerations in pharmacogenetic testing.	CYP2C9	ADRB2	DRD2	VKORC1	HLA-B				
(2014) Genomic architecture of pharmacological efficacy and adverse events.	CYP2C9	COMT	DRD2	VKORC1	HLA-B				
(2014) An ontology-based, mobile-optimized system for pharmacogenomic decision support at the point-of-care.	CFTR	CYP2C9	EGFR	VKORC1	HLA-B				
(2012) Systems pharmacology: network analysis to identify multiscale mechanisms of drug action.	CYP2C9	ADRB2	COMT	VKORC1	GSTP1				

Figure S1. Visualizing Reference Publications (2022 - 2012) about pharmacogenomic with VIPs and non-VIP proteins. The figure integrates key pharmacogenomic literature with molecular findings. The Lollipop plot correlates Reference Publication from PubMed Enrichment (X-axis) with -log_{10}(FDR) (Y-axis) and gene count (node size). The adjacent panel maps each publication to a curated set of Matching Proteins from Network. Protein names highlighted in red (EGFR and MYC) are classified as Non-VIP (Non-Very Important Protein), distinguishing them from core VIP network members.
